# Platypnea-Orthodeoxia Syndrome: A Rare Cause of Positional Respiratory Failure

**DOI:** 10.7759/cureus.32538

**Published:** 2022-12-14

**Authors:** Pedro R Soares, Nuno Melo, Diana Ferrao, Estela Sousa, Adriana Santos, André Gomes, Fernando Friões

**Affiliations:** 1 Internal Medicine Department, Centro Hospitalar Universitário de São João, Porto, PRT; 2 Intermediate Care Unit, Internal Medicine Department, Centro Hospitalar Universitário de São João, Porto, PRT

**Keywords:** atrial septal defects occluder, positional dyspnea, hypoxemic respiratory failure, patent foramen ovale, platypnea-orthodeoxia syndrome

## Abstract

Platypnea-orthodeoxia syndrome (POS) is a rare clinical entity characterized by dyspnea and arterial desaturation in the upright position. Hypoxia in POS has been attributed to the mixing of deoxygenated venous with oxygenated arterial blood via a shunt, with patent foramen ovale being the most commonly reported abnormality. A systematic evaluation is necessary to identify the underlying cause and promote an appropriate intervention. Here, we present the case of a 79-year-old female with a new diagnosis of POS during the workup of hypoxemic respiratory failure.

## Introduction

Platypnea-orthodeoxia syndrome (POS) is characterized by positional dyspnea (platypnea) and arterial desaturation while in the upright position (orthodeoxia). The definition of orthodeoxia denotes a drop in saturation of >5% from a supine to an upright position [1]. Since the first case, described in 1949 by Burchell et al. in a patient with post-traumatic intrathoracic arteriovenous shunt [2], less than 190 cases have been reported [3].

Although the pathophysiology of POS has puzzled clinicians for years and in some patients the precise mechanisms remain elusive [1], there are three acknowledged etiologies, namely, cardiac conditions with intracardiac shunting, pulmonary diseases with ventilation/perfusion mismatch, and hepatic diseases with pulmonary arteriovenous shunts [4,5]. Considering all potential etiologies for this syndrome, intracardiac abnormalities are the most frequently encountered, with patent foramen ovale (PFO) being the most common cause of intracardiac shunt, followed by atrial septal defects and atrial septal aneurysms [4,6]. Here, we present the case of a 79-year-old female with POS who was diagnosed during the workup of hypoxemia respiratory failure.

## Case presentation

We report the case of a 79-year-old female with a previous history of asthma, hypertension, and cerebrovascular disease. She was admitted to the Emergency Department with progressive dyspnea, dry cough, and an episode of lipothymia while sitting. On admission, she was hemodynamically stable (blood pressure of 126/78 mmHg, heart rate of 79 beats per minute), peripheral oxygen saturation was 76% while breathing room air, respiratory rate was 28 cycles per minute, and she was febrile (temperature of 38.2°C). The remaining physical examination was unremarkable except for the presence of a holosystolic heart murmur at the left sternal border. An arterial blood sample revealed a hypoxemic respiratory failure (with a partial pressure arterial oxygen/fraction inspired oxygen ratio of 200). Additional blood tests revealed mild leukocytosis and elevated C-reactive protein (105 mg/L; normal value <3.0 mg/L). Electrocardiogram revealed a sinus rhythm without signs of acute or chronic ischemia. Computed tomography (CT) of the chest showed an ectasia of the ascending aorta, but no findings of pulmonary embolism, effusions, or consolidations were noted. Amoxicillin/clavulanic acid was started for a suspected respiratory infection, and the patient was admitted to the Internal Medicine ward.

**Figure 1:**
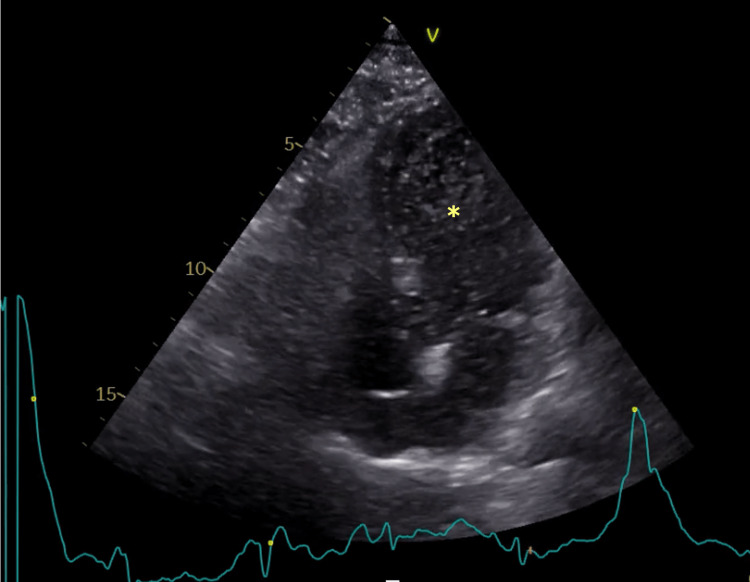
Agitated saline test during a transthoracic echocardiogram. A positive agitated saline/bubble test was observed during the transthoracic echocardiogram, showing bubbles in the left side of the heart (* – left ventricle).

**Figure 2:**
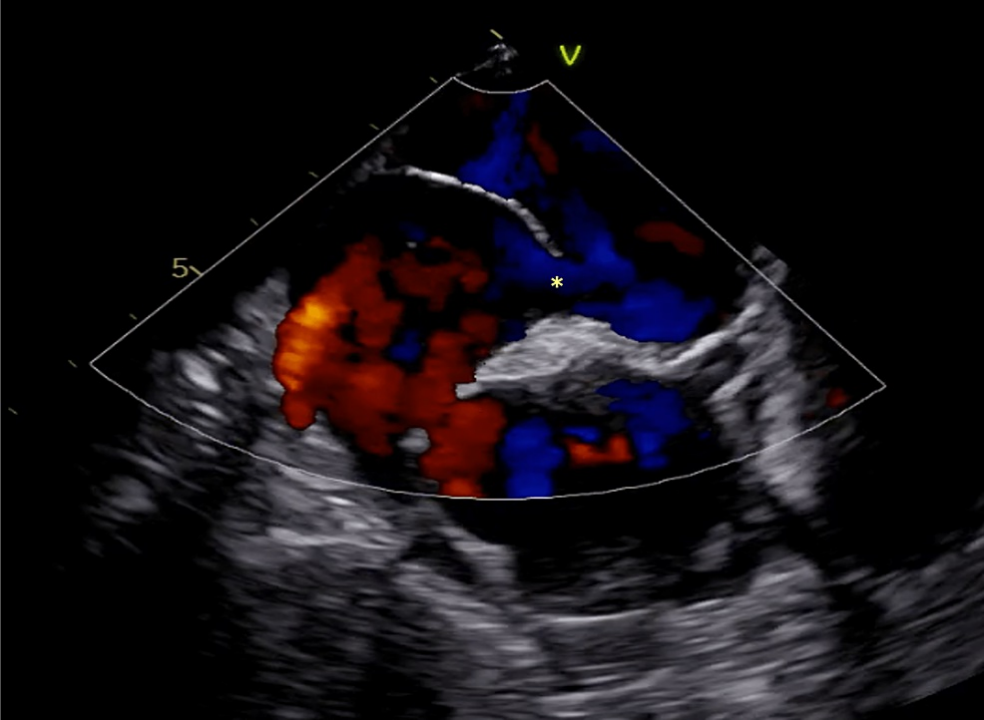
Transesophageal echocardiogram. A large patent foramen ovale (*) with a bidirectional shunt was noted in the transesophageal echocardiogram.

After five days of amoxicillin/clavulanic acid, she became apyretic, and a decline in inflammatory markers was noted. Conversely, no improvement in hypoxemic respiratory failure was observed. A transthoracic echocardiogram (TTE) was performed which described a preserved left systolic function and an absence of major valvular abnormalities. During hospitalization, an episode of lipothymia and arterial desaturation while sitting was observed and promptly resolved after assuming a recumbent position. A subsequent agitated saline TTE was immediately positive (Figure 1), with the presence of bubbles in the left heart within three beats, suggestive of an intracardiac shunt. A transesophageal echocardiogram corroborated the findings, revealing a large PFO with a bidirectional shunt (Figure 2). Additionally, further tests were negative for lung ventilation/perfusion mismatch or liver disease.

After the diagnosis of POS in the setting of PFO, the patient underwent a percutaneous correction of the intracardiac shunt with an Amplatzer™ Atrial Sept Defect Occluder. Immediately after the procedure, a dramatic improvement in respiratory failure was noted and the patient was discharged one week after on supplementary oxygen. At three months after discharge, the patient experienced marked symptomatic relief, including complete resolution of the hypoxemic respiratory failure. A subsequent TTE after PFO closure was performed, showing the correct placement of the Amplatzer™ device and a small residual shunt (Video 1).

**Video 1:** Transthoracic echocardiogram after patent foramen ovale closure. A transthoracic echocardiogram was performed after patent foramen ovale closure with an Amplatzer™ device showing minimal residual shunt.

## Discussion

The prevalence of POS is probably underestimated, making its awareness particularly significant. It is essential to have a high suspicion to detect this syndrome in patients with dyspnea, given the subtle and positional nature of the symptoms [1].

Although the pathophysiology of POS remains unsettled, two conditions must coexist to diagnose the syndrome, namely, an anatomical component (intracardiac shunt, a ventilation/perfusion mismatch, or pulmonary arteriovenous shunts) and a functional component that promotes the redirection of shunt flow with the assumption of an upright posture [7,8]. The former may be an atrial septal defect or a PFO, while the latter may be cardiac (pericardial effusion or constrictive pericarditis), pulmonary (emphysema, pneumectomy, or amiodarone toxicity), abdominal (hepatic cirrhosis or an ileus), or vascular (an aortic aneurysm or an elongation) [7].

The functional component is intriguing. The prevalence of PFO may be as high as 30% in some series, although most patients remain asymptomatic [4,9]. Therefore, despite PFO being considered one of the most common anatomic defects underlying POS, its presence is not sufficient for the development of the syndrome. In this case, the anatomic component is evident, an intracardiac shunt caused by a PFO. On the contrary, the functional component is arguable despite extensive investigation. We hypothesized that the observed aortic elongation could be a potential mechanism for the development of POS in this patient, as previously described [7].

A contrast-enhanced transesophageal echocardiogram at rest and during the Valsalva maneuver is considered the gold standard for diagnosis [4]. In the presence of symptoms attributed to the PFO, as in POS, the atrial defect may be treated with percutaneous closure [1,4]. The recognition of this entity is essential during the approach of a patient with positional dyspnea, as the correct treatment can alleviate symptoms and be potentially curative.

## Conclusions

The prevalence of POS in recent literature remains rare. Two conditions must co-exist to cause this syndrome: an anatomical component, such as intertribal communication, and a functional component that provokes the redirection of shunt flow in an upright posture, such as an aortic aneurysm or elongation. Despite the high prevalence of PFO, the emergence of POS in this group of patients is scarce, and the explanation for this phenomenon remains to be deciphered. POS is an under-recognized clinical entity, with intracardiac shunting being the most common mechanism, particularly a PFO. A high level of suspicion is the cornerstone to a definitive diagnosis and respective treatment for patients presenting with this intriguing syndrome.

## References

[1] Agrawal A, Palkar A, Talwar A (2017). The multiple dimensions of platypnea-orthodeoxia syndrome: a review. Respir Med.

[2] Burchell HB, Wood EH (1949). Reflex orthostatic dyspnea associated with pulmonary hypotension. Am J Physiol.

[3] Rodrigues P, Palma P, Sousa-Pereira L (2012). Platypnea-orthodeoxia syndrome in review: defining a new disease?. Cardiology.

[4] Queirós C, Francisco E, Almeida J (2017). Platypnea-orthodeoxia syndrome after complicated cholecystectomy: an unsuspected diagnosis. Acta Med Port.

[5] Akin E, Krüger U, Braun P (2014). The platypnea-orthodeoxia syndrome. Eur Rev Med Pharmacol Sci.

[6] Knapper JT, Schultz J, Das G, Sperling LS (2014). Cardiac platypnea-orthodeoxia syndrome: an often unrecognized malady. Clin Cardiol.

[7] Cheng TO (2002). Mechanisms of platypnea-orthodeoxia: what causes water to flow uphill?. Circulation.

[8] Cheng TO (1999). Platypnea-orthodeoxia syndrome: etiology, differential diagnosis, and management. Catheter Cardiovasc Interv.

[9] Hagen PT, Scholz DG, Edwards WD (1984). Incidence and size of patent foramen ovale during the first 10 decades of life: an autopsy study of 965 normal hearts. Mayo Clin Proc.

